# The genome-scale metabolic model for the purple non-sulfur bacterium *Rhodopseudomonas palustris* Bis A53 accurately predicts phenotypes under chemoheterotrophic, chemoautotrophic, photoheterotrophic, and photoautotrophic growth conditions

**DOI:** 10.1371/journal.pcbi.1011371

**Published:** 2023-08-09

**Authors:** Diego Tec-Campos, Camila Posadas, Juan D. Tibocha-Bonilla, Deepan Thiruppathy, Nathan Glonek, Cristal Zuñiga, Alejandro Zepeda, Karsten Zengler

**Affiliations:** 1 Facultad de Ingeniería Química, Universidad Autónoma de Yucatán, Mérida, Yucatán, México; 2 Department of Pediatrics, University of California, San Diego, La Jolla, California, United States of America; 3 Bioinformatics and Systems Biology Graduate Program, University of California, San Diego, La Jolla, California, United States of America; 4 Department of Bioengineering, University of California, San Diego, La Jolla California, United States of America; 5 Center for Microbiome Innovation, University of California, San Diego, La Jolla, California, United States of America; Center for Advanced Systems Understanding (CASUS), GERMANY

## Abstract

The purple non-sulfur bacterium *Rhodopseudomonas palustris* is recognized as a critical microorganism in the nitrogen and carbon cycle and one of the most common members in wastewater treatment communities. This bacterium is metabolically extremely versatile. It is capable of heterotrophic growth under aerobic and anaerobic conditions, but also able to grow photoautotrophically as well as mixotrophically. Therefore *R*. *palustris* can adapt to multiple environments and establish commensal relationships with other organisms, expressing various enzymes supporting degradation of amino acids, carbohydrates, nucleotides, and complex polymers. Moreover, *R*. *palustris* can degrade a wide range of pollutants under anaerobic conditions, e.g., aromatic compounds such as benzoate and caffeate, enabling it to thrive in chemically contaminated environments. However, many metabolic mechanisms employed by *R*. *palustris* to breakdown and assimilate different carbon and nitrogen sources under chemoheterotrophic or photoheterotrophic conditions remain unknown. Systems biology approaches, such as metabolic modeling, have been employed extensively to unravel complex mechanisms of metabolism. Previously, metabolic models have been reconstructed to study selected capabilities of *R*. *palustris* under limited experimental conditions. Here, we developed a comprehensive metabolic model (M-model) for *R*. *palustris* Bis A53 (*i*DT1294) consisting of 2,721 reactions, 2,123 metabolites, and comprising 1,294 genes. We validated the model using high-throughput phenotypic, physiological, and kinetic data, testing over 350 growth conditions. *i*DT1294 achieved a prediction accuracy of 90% for growth with various carbon and nitrogen sources and close to 80% for assimilation of aromatic compounds. Moreover, the M-model accurately predicts dynamic changes of growth and substrate consumption rates over time under nine chemoheterotrophic conditions and demonstrated high precision in predicting metabolic changes between photoheterotrophic and photoautotrophic conditions. This comprehensive M-model will help to elucidate metabolic processes associated with the assimilation of multiple carbon and nitrogen sources, anoxygenic photosynthesis, aromatic compound degradation, as well as production of molecular hydrogen and polyhydroxybutyrate.

## Author summary

*Rhodopseudomonas palustris* actively participates in the carbon and nitrogen cycles by fixing carbon dioxide (CO_2_) and molecular nitrogen (N_2_), as well as transforming nitrate into N_2_. This photosynthetic bacterium has been extensively studied and is considered a model microorganism for elucidating carbon and nitrogen fixation, H_2_ production, denitrification, aromatic compounds degradation, anoxygenic photosynthesis and the production of complex polymers like polyhydroxyalkanoates. Constraint-based metabolic models are systems biology tools capable of predicting an organism’s phenotype under several growth conditions. The metabolic model of *R*. *palustris* Bis A53, *i*DT1294, accurately predicts growth rates of over 350 carbon and nitrogen sources (e.g. amino acids, aromatic compounds, organic acids, nucleotides, etc.) under different oxygen and light conditions. Furthermore, *i*DT1294 precisely simulates how *R*. *palustris* switches between photoheterotrophic and photoautotrophic metabolic modes over time, depending on nutrient availability and light conditions. The model represents the most comprehensive M-model for a purple non-sulfur bacterium to date, thus aiding the elucidation of *R*. *palustris’* metabolic roles in different environments.

## 1. Introduction

*Rhodopseudomonas palustris* is a photosynthetic Gram-negative purple non-sulfur bacterium (PNSB) of the family *Bradyhizobiaceae*. It can attain a wide range of metabolic states and is considered one of the most versatile microorganisms [1]. *R*. *palustris’* "Swiss army knife metabolism" renders it capable of utilizing a broad range of substrates [2]. This facultative PNSB can grow under aerobic or anaerobic conditions [3,4], activating oxygen-sensitive regulation strategies related to nitrogen fixation, denitrification, aromatic compound degradation and polyhydroxybutyrate (PHB) metabolism [5,6]. In addition to its flexibility in regard to oxygen levels, it can utilize various carbon and nitrogen sources [1,3,4,7] and is capable of assimilating both organic (heterotrophy metabolism) as well as inorganic (autotrophy metabolism) compounds [4,8–11]. The list of organic compounds assimilated by *R*. *palustris* includes amino acids, organic acids, carbohydrates, aromatic compounds, and highly complex polymers like plant-derived biomass [12–18]. At the same time, this bacterium possesses highly specialized enzymes for autotrophic growth, encoding genes for form I and form II of the rubisco enzyme [19–24]. *R*. *palustris* is also a diazotroph, capable of fixing molecular nitrogen (N_2_) using three highly specialized metal- (iron, vanadium, and molybdenum) dependent nitrogenases [8,25]. The activity of these nitrogenases is highly susceptible to mineral and molecular oxygen (O_2_) concentrations and expression of these enzymes is thus highly regulated based on O_2_ availability [26]. Nitrogen fixation to ammonium in *R*. *palustris* is linked to the production of molecular hydrogen (H_2_), an aspect of interest to the alternative fuel sector [14,15,27,28]. Additional to nitrogen fixation, *R*. *palustris’* nitrogen metabolism capabilities include the transformation of nitrate into N_2_ [29,30]. Under stress conditions *R*. *palustris* not only produces N_2_ from this reaction but also byproducts such as nitrite, as well as nitric and nitrous oxides [2]. Besides the metabolic flexibility for carbon and nitrogen consumption, *R*. *palustris* generates energy (ATP) under anoxic conditions by either anoxygenic photosynthesis or by denitrification using nitrate as electron acceptor [29–31]. Hence, this extremely versatile bacterium can grow in four distinct modes, i.e., chemoheterotrophy, chemoautotrophy, photoautotrophy, and photoheterotrophy [32–35].

The metabolic flexibility of *R*. *palustris* has rendered this bacterium a prime candidate for the production of high-value compounds [6,36,37]. Specifically, the potential of *R*. *palustris* for the degradation of pollutants have spurred interest in wastewater recycling and soil bioremediation [38–40]. Additionally, the production of different polyhydroxyalkanoates and PHBs by this PNSB is of interest to the bioplastic industry [28,41,42] The production of H_2_ by *R*. *palustris* has been explored in the past as bioenergy alternative [4,43]. Due to these diverse metabolic features, it is not surprising that *R*. *palustris* can be found in various ecosystems and microbial communities [44–46]. *R*. *palustris* is present in aquatic sediments, wastewater microbial communities, contaminated soils, and can interact and form relationships with plants [44,47,48] and other microorganisms [35,49,50]. Several metabolic strategies of *R*. *palustris* Bis A53 have been previously unraveled, but there are still unknown metabolic features and mechanisms related to the carbon and nitrogen assimilation employed by bacterium.

Previous studies in which metabolic models for *R*. *palustris* were successfully employed provided new knowledge about the metabolic mechanisms and fluxes of its growth under anoxic conditions in the light [51,52]. However, these studies focused mainly on phototrophic phenotypes of *R*. *palustris*, omitting a wide range of this bacteria’s possible lifestyles. Here we applied a systems biology approach to predict *R*. *palustris* Bis A53 metabolic fluxes under a variety of different experimental conditions at the genome-scale. A whole-genome perspective of the metabolism provides a complete picture of active pathways as well as metabolic fluxes. Genome-scale metabolic models (GEMs) present high-quality predictive performances when semi-automated tools are utilized for the reconstruction process [26,53–55]. The resulting draft model of *R*. *palustris* Bis A53 was manually curated using available data from several bioinformatics databases. We utilized experimental data from the literature and performed additional experiments to fine-tune metabolic constraints under the four metabolic modes of *R*. *palustris*. We compiled all data and validated the model’s growth rate predictions from Flux Balance Analysis (FBA) under different conditions. A wide range of varied carbon and nitrogen sources were evaluated and the precision of the model to predict the vast metabolism of *R*. *palustris* was determined. The model accuracy was compared to other automatic and curated models. Ultimately, the model was validated using kinetic experiments from literature evidence to determine *i*DT1294 robustness using dynamic Flux Balance Analysis (dFBA) [56]. The M-model accurately predicted the metabolic changes between photoheterotrophic and photoautotrophic conditions over time.

## 2. Results

### 2.1. Metabolic network reconstruction of *R*. *palustris* Bis A53

**Fig 1:**
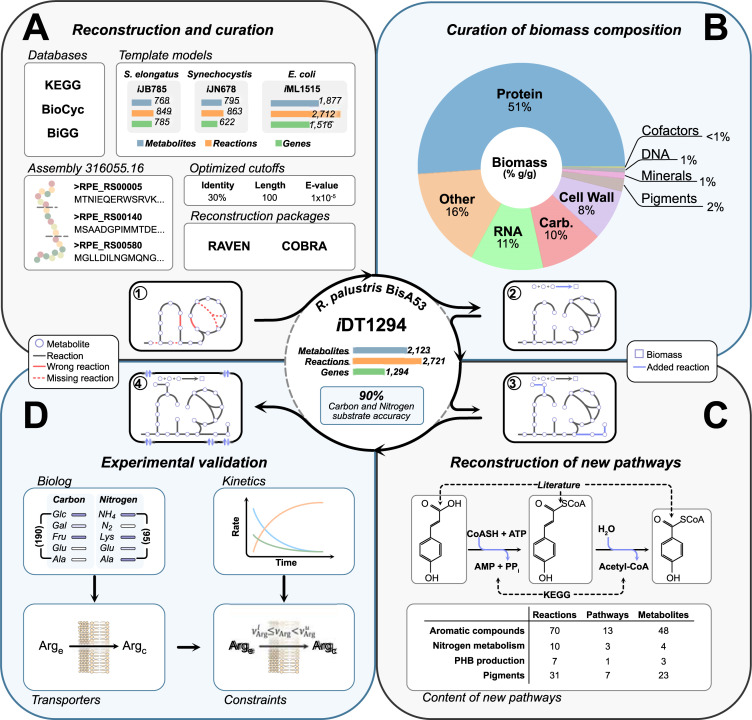
Workflow to build a metabolic model of *R*. *palustris* BisA53 using a semiautomatic approach. **A** A draft model was reconstructed using optimized BLAST parameters (e-value, query length, and identity percentage) from three template models present in BiGG (*Escherichia coli* K-12 substr. MG1655, *Synechocystis* sp. PCC 6803, and *Synechococcus elongatu*s PCC 7942). NCBI reference sequence annotation was employed in GPR associations. The RAVEN and COBRA packages for MATLAB were deployed in the reconstruction stage. **B** The resulting optimized draft model and constituents of the Biomass Objective Function (BOF) were manually curated. Protein, RNA and DNA metabolites of the BOF were calculated based on genomic and proteomic data, meanwhile the rest of the metabolic requirements were estimated based on experimental evidence previously published. Disconnected metabolites were properly integrated into metabolic pathways using bioinformatics databases. The *i*DT1278 model (*A*. *vinelandii*) worked as a template for nitrogen fixation, hydrogen production, and PHB biosynthesis reactions using BLASTp homology. The resulting draft model of the BOF and dead-ends curation contained 2,298 reactions, 1,918 metabolites, and 1,515 genes (200 exogenous genes). **C** Four detailed subsystems were manually added to the model to reflect specific metabolic capabilities of *R*. *palustris* BisA53: aromatic compound degradation under anaerobic conditions, nitrogen fixation and denitrification (with partial denitrification), PHB production and pigment metabolism (including carotenoid and bacteriochlorophyll of PNSB). For the aromatic compounds biosynthesis pathways, the reactions and metabolites involved were carefully added based on enough experimental evidence and curated information of the pathways from the bioinformatics databases (See Methods). **D** The resulting model was validated using experimental data retrieved from the literature and growth experiments performed in this study. The iterative model refinement process included manual curation, gap-filling, and curation under chemoheterotrophic, chemoautotrophic, photoautotrophic, and photoheterotrophic conditions changing the oxygen requirements depending on the experimental environments. Ultimately, the model was validated using kinetics data to compare the prediction capabilities of the M-model using growth rates and substrate concentrations for specific time points. The final model, containing 2,721 reactions, 2,123 metabolites, and 1,294 genes, predicted growth with 90% accuracy for carbon and nitrogen substrates.

We used semiautomatic strategies to reconstruct the M-model of *R*. *palustris* Bis A53. This approach has been successfully applied in the past to reconstruct bacterial M-models [53]. First, an initial draft model of *R*. *palustris* Bis A53 was created using the genome annotation of the NCBI Reference Sequence database: GCF_000014825.1. Four manually curated and previously validated M-Models were used as protein homology templates: *Synechocystis sp*. PCC 6803 (*i*JN678) [57], *Synechococcus elongatus* PCC 7942 (*i*JB785) [58], *Escherichia coli* K-12 substr. MG1655 (*i*ML1515) [59], and *Azotobacter vinelandii* DJ (*i*DT1278) [26]. The reference models were employed in different stages of the reconstruction process to carefully identify homology of the subsystems and properly distribute metabolites across the compartments. The M-models of the photosynthetic microorganisms (*Synechocystis sp*. PCC 6803 and *Synechococcus elongatus* PCC 7942) and *i*ML1515 (*E*. *coli*) were utilized to determine the optimal cut-off parameters (e-value, query length, and percentage of identity) for the BLAST algorithm to reconstruct the initial draft M-model (Fig 1A). The RAVEN and COBRA Toolboxes [56,60] were used to generate the semiautomatic draft reconstruction. A total of 384 draft models were generated varying the three BLAST parameter criteria (S1 Material) to evaluate the effect of each BLAST parameter and their interaction in the model reconstruction phase. The range of values tested for the three variables was: e-value (1x10-30-1 x10-5), query length (50–150 amino acids), and percentage of identity (20–40%). A set of 391 reactions and their gene-protein-reaction (GPR) associations were manually curated to use as a quality control check of the GPR associations automatically determined by the metabolic modeling toolboxes. Five principal variables were calculated based on the results of the quality of each resulting model: (1) incorrect homology calls included in GPR associations of the draft model (false positive accumulative); (2) sum of genes not assigned (missing genes) in the GPR associations (false negative accumulative); (3) unique genes incorrectly contained in the rules of draft models determined from false positive accumulative (unique false positives); (4) unique missing genes involved in rules of resulting models (unique false negatives); and (5) the entirety of genes correctly assigned in GPR associations (true positive accumulative). As expected, relaxed values of the three BLAST parameters increased the number of true positive calls in GPR associations. For instance, one of the draft models generated (using e-value = 1x10-5, query length = 50 aa, and identity percentage = 20%) contained almost 350 genes correctly assigned across the 391 reactions (higher amount of hits than 99% of the draft models reconstructed). However, incorrect homology calls dramatically increased under this criterion (false positive accumulative = 1650 and unique false positives = 623). More restricted values decreased the number of false positive calls, sacrificing the total of true positive calls (correlated to the number of false negative calls). Based on these preliminary results, we focused on draft models with average criteria (specifically for query length and identity percentage) since most of these initial models contain a considerable number of hits (close to 300) and significantly fewer unique false positive and negatives (50% less than draft models with relaxed criteria). From a second stage of BLAST parameters screening, 10 models were chosen as suitable draft models based on false positive and negative calls. The GPR associations of these draft models were carefully reviewed to identify which kind of reactions and associations were wrongly assigned. A final draft model was selected with the following parameters: e-value = 1x10-5, query length = 100 aa, and identity percentage = 30%. Similar values have been successfully employed in semiautomatic reconstruction strategies for other template microorganisms [26,54,55,61]. However, the resultant optimal BLAST cut-offs have never been estimated for photosynthetic bacteria. Each reaction of the optimized draft model was evaluated for free energy production (ATP, NADH, and NADPH accumulation) and mass balances as part of the quality control tests to guarantee model functionality and chemical accuracy. Reactions associated with template genes were maintained in the draft model to guarantee model connectivity across compartments and the capability of the model to perform growth simulations. The resulting optimized draft model from three core templates contained 1,732 metabolic reactions and 1,569 metabolites divided into five compartments (cytoplasm, periplasm, carboxysome, thylakoid, and extracellular space) with a total of 1,398 genes (208 exogenous genes from the templates). Nitrogen fixation, H_2_ consumption, and PHB biosynthesis reactions were imported from *i*DT1278 (*A*. *vinelandii*) in the model refinement stage with the GPR adjustment using *R*. *palustris* homologous proteins.

#### 2.1.1. Model refinement

The model refinement was executed following manual curation and gap filling. Every GPR association created during the draft model stage was investigated and validated by comparing the predicted GPR association based on BLAST to the annotation of the genes from different bioinformatic databases (e.g., KEGG[62], BioCyc[63], BRENDA[64], UniProt[65], and MetaNetX[66]) as well as available biochemical information from the literature (See Methods). Manual curation was based on protein sequence homology and EC number annotations. Additionally, the new validated GPR associations were cross-checked using a secondary assessment based on NCBI and PATRIC annotations. For GPR associations with exogenous proteins, multiple protein sequences were aligned using *R*. *palustris* Bis A53 proteome and proteins from the BiGG database [67] associated with those reactions. *R*. *palustris* amino acid sequences (RPE) aligned under the selected BLASTp parameters (see Methods) were manually verified based on the information in the databases and assigned to the corresponding reactions. Reactions without associations to *R*. *palustris* were identified and evaluated using sink metabolite algorithms [56]. Reactions with exogenous GPR associations were classified according to their flux contribution to the BOF and their biological relevance. The tested reactions with no contribution to the BOF and without biological relevance were removed from the model. Reactions involved in the production of BOF components or with biological significance were kept in the M-model and designated as orphan reactions (S6 Material).

**Fig 2:**
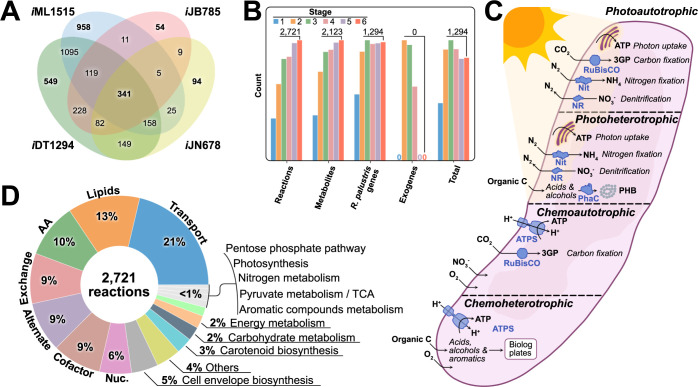
Model properties and prediction capabilities of iDT1294. **A** A general comparison was executed among the three principal template models (*Escherichia coli* K-12 substr. MG1655, *i*ML1515, *Synechocystis* sp. PCC 6803, *i*JN678 and *Synechococcus elongatus* PCC 7942, *i*JB785) and *i*DT1294 reactions. The four models share 341 core metabolic reactions. **B** Six principal model versions were generated from the initial reconstruction process to the final validated model. Across the different stages, the model increased the number of reactions, metabolites, and RPE genes, while the number of exogenous genes was reduced to zero. **C** *i*DT1294 accuracy and phenotypic predictions capabilities were evaluated under the four main metabolic states of this versatile organism, testing over 350 experimental conditions. **D** *R*. *palustris* Bis A53 model contains 549 unique reactions related to aromatic compounds metabolism, PHB production, carotenoids, pigments and bacteriochlorophylls biosynthesis specifically synthetized by PNSB as well as nitrogen fixation and denitrification. Reactions (2,721 in total) were distributed in 17 subsystems representing the entire metabolism of *R*. *palustris* Bis A53.

The preliminary draft model with optimized BLASTp parameters (only containing *i*JB785 and *i*JN678 as references) consisted of 956 reactions, 802 metabolites, and 725 RPE genes. The draft model increased to 1,732 reactions, 1,569 metabolites, and 1,398 genes (1,198 of which had amino acid sequence information, i.e., RPE genes) after adding the homologous metabolic properties from *i*ML1515 using the RAVEN toolbox [60]. Subsequently, the *R*. *palustris* GEM from the first manual curation stage was complemented using another metabolic model from the CarveMe prokaryotic modeling database [68]. Both metabolic models were compared based on reactions, metabolites, and genes involved in each model. The reactions only present in the CarveMe version were manually verified (GPR associations) and added to the draft model, checking energy and mass balances. A total of 566 reactions, 349 metabolites, and 117 genes were added to the semi-curated M-model (Fig 2). Prior to the gap-filling stage, all transport reactions of the draft model were curated using TransportDB [69] and metabolites were assigned to three compartments (cytosol, periplasm, and extracellular space). Reactions and metabolites that were originally located in the thylakoid and carboxysome compartment of the template models (neither of them is present in *R*. *palustris*) were reassigned to the cytosol and periplasm to maintain the biological relevance of the model. Carboxysome and thylakoid reactions were reallocated based on KEGG and BioCyc annotations regarding *R*. *palustris*’ photosynthetic pathway distribution. This step resulted in a model comprised of 2,349 reactions, 1,955 metabolites, and 1,275 genes distributed across three compartments.

#### 2.1.2. Gap filling

Gap filling was executed in two separate steps: (1) gap filling of disconnected metabolites already present in the model and (2) gap filling based on experimental results, which consisted of (2a) phenotyping using Biolog plates and (2b) previously published studies. Initially, disconnected metabolites of the manually curated draft model were determined using COBRA Toolbox algorithms [56]. The disconnected metabolites were classified into three groups (see Methods) and reconnected depending on available information in bioinformatics databases. Metabolites remaining from template models but without evidence being present in *R*. *palustris* were removed together with their associated reactions. During the second step of gap filling, we used experimental data from Biolog experiments (PM1 and PM2 plates for carbon sources and PM3 for nitrogen sources) and modified reactions in the model accordingly. Each reaction added to the model in this step was manually reviewed to maintain concordance in the GPR associations, and energy and mass balance curation was performed to preserve the quality of the model. Transport reactions involved in the assimilation of carbon and nitrogen sources from Biolog results were associated with RPE genes using specific transporter information of the NCBI and PATRIC annotations. Unspecific transporting proteins were assigned to carbon and nitrogen sources without detailed information on the proteins implicated in inter- or intracelluar transport (e.g., general ABC amino acids transporters, general symporters, antiporters, porins, etc.). Furthermore, metabolic reactions involved in the catabolism of carbon and nitrogen sources revealed by Biolog experiments were added to the model with the corresponding RPE genes in GPR associations. Reactions employed in the suitable assimilation of the tested compounds without RPE evidence were kept in the model as orphan reactions. The resultant gap filled model contained 2,655 reactions, 2,081 metabolites and 1,282 RPE genes. In the second subsection of the final gap filling stage, literature information was deployed using an iterative approach to determine which reactions were missing in the model for their proper consumption. The model was modified by adding pathways for aromatic compound degradation and their use as carbon source under photoheterotrophic conditions. Further additions included pathways for nitrogen fixation and denitrification (nitrate and nitrite). In Fig 1C, the new relevant pathways added to the model are summarized and classified according to the pathways, reactions, and metabolites involved. Overall, a total of 372 reactions and 168 metabolites across all compartments were added to the final model during the gap-filling process.

#### 2.1.3. Model properties

The *R*. *palustris* Bis A53 metabolic model (*i*DT1294) consists of 2,721 metabolites, 2,123 reactions, and 1,294 genes, representing ~27% of all annotated coding genes in the NCBI genome reference sequence. Specific details about the reactions and metabolites from the model are summarized in Fig 2. *i*DT1294 was validated using over 350 experimental growth results under photoheterotrophic, photoautotrophic, nitrogen fixing (diazotroph), and heterotrophic conditions (both aerobically and anaerobically). *i*DT1294 contains all reactions, metabolites, and genes involved in nitrogen fixation and denitrification (nitrogen metabolism), PHB production, carotenoid and bacteriochlorophyll biosynthesis (S4 Material), anoxygenic photosynthesis, and aromatic compound degradation (Fig 2B). The entirety of the metabolic pathways present in the M-model is organized in 17 subsystems (including transport and exchange reactions) depending on the biological role of the reactions and metabolites utilized (Fig 2).

**Fig 3:**
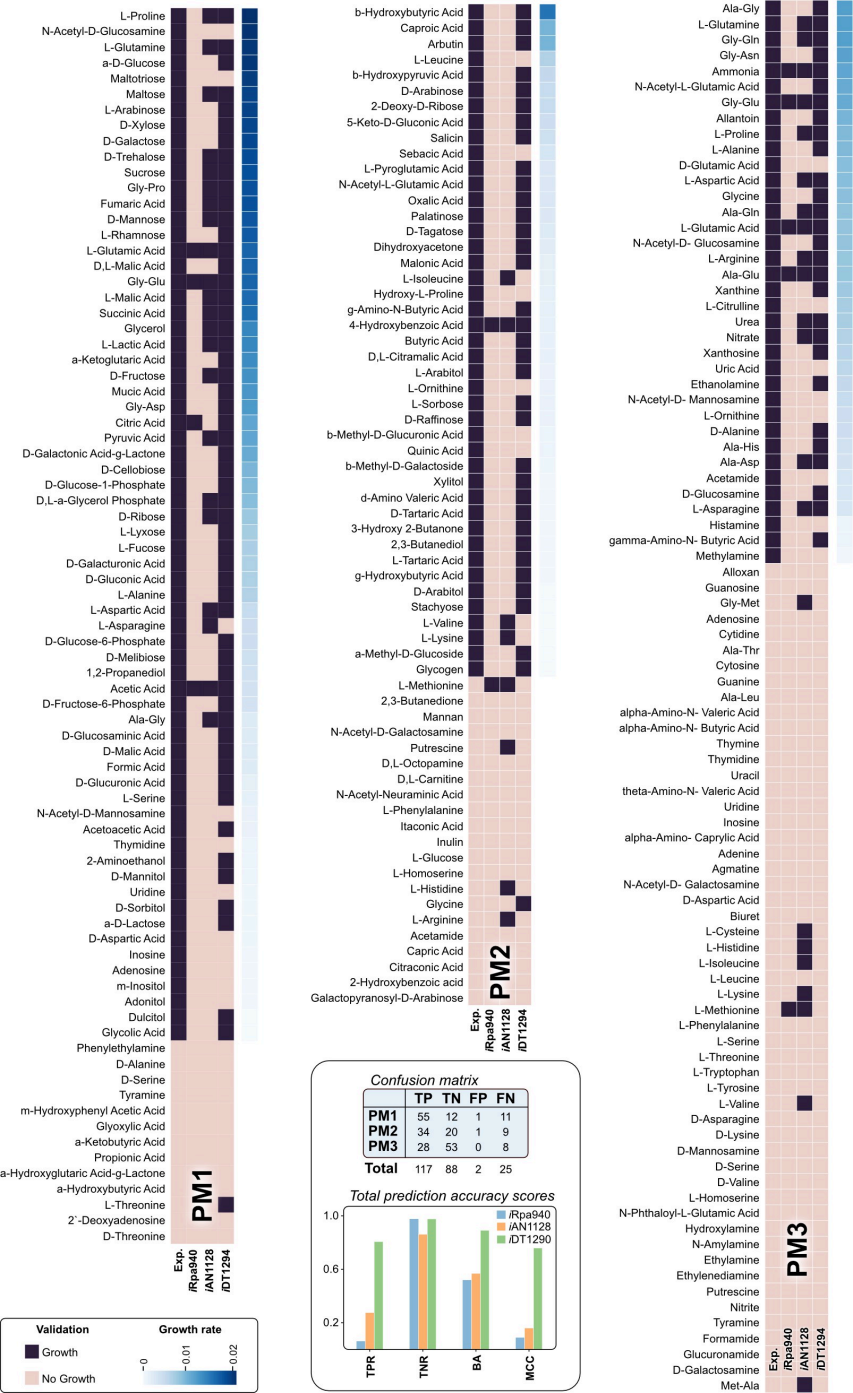
Comparison of the high-throughput growth phenotypic experimental and simulated results across the three principal metabolic models. *R*. *palustris* Bis A53 was cultivated in 190 carbon and 95 nitrogen sources under monoculture conditions for 96 hours. Subsequently, the three GEMs (*i*DT1294, *i*Rpa940, and *i*AN1128) were properly constrained to simulate all the Biolog conditions. Each heat map compares every carbon (PM1 and PM2) and nitrogen (PM3) source’s experimental result against the simulation growth output per metabolic model. Growth results were classified into two possible results: Growth (purple) and No Growth (light pink). Additionally, we determined growth by measuring optical density (OD600, see methods) after 96 hrs, as indicated in blue to the right of each Biolog plate set. The substrates studied were sorted in descending order based on growth values. Statistical parameters to determine the global accuracy and prediction capabilities of the three models were calculated.

**Fig 4:**
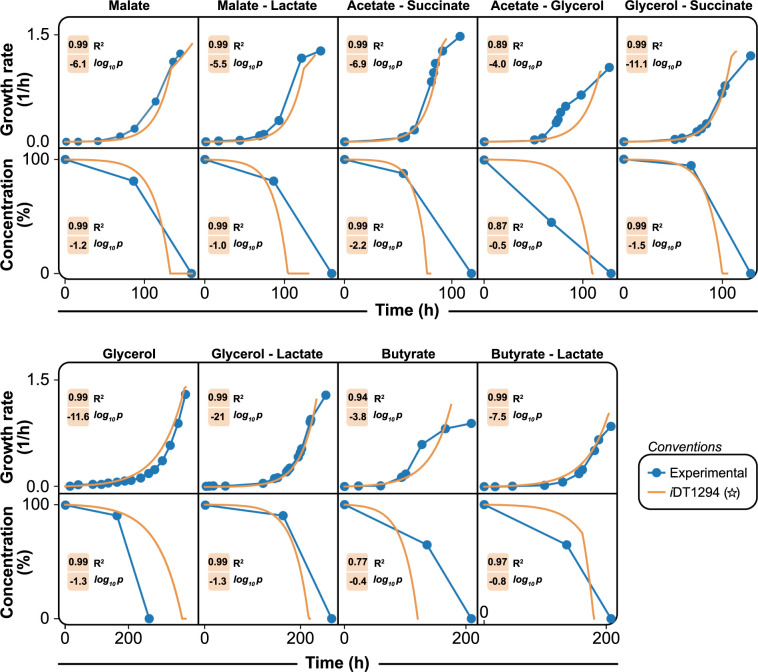
Comparison of dynamic flux balance analysis with experimental data. The set of plots shows the dynamic prediction capabilities of *i*DT1294 using nine different growth conditions. For each plot, the experimental time points (blue dots) were adjusted to the exponential phase, and time points in the stationary phase were removed. Later, dFBA was executed for *i*DT1294 to determine the growth rates and consumption of substrates across time. R2 and log_10_P parameters were determined for *i*DT1294 (orange and star) to identify how the model predictions fit to the experimental results.

Most of the reactions (80%) in *i*DT1294 belong to lipid metabolism, amino acid metabolism, transport and exchange of metabolites, alternate carbon and cofactor, and vitamin metabolism. Specific metabolic capabilities of *R*. *palustris* Bis A53, such as nitrogen fixation and denitrification (nitrogen metabolism), PHB production (others), carotenoids metabolism (including pigments and bacteriochlorophylls biosynthesis), anoxygenic photosynthesis and aromatics compound degradation represent less than 6% of the metabolic reactions. Through this metabolic pathways’ distribution, *i*DT1294 can predict all four metabolic modes of *R*. *palustris* accurately (Figs 3 and 4). The three principal template models used during the optimization and reconstruction steps share 341 reactions with *i*DT1294. Most of the reactions present in the four models are related to core metabolic pathways (TCA cycle, glycolysis, gluconeogenesis, amino acids, lipids, carbohydrates and cofactor metabolism). Photosynthesis, carotenoid and pigments biosynthesis, and some alternate carbon pathways were taken from the two photosynthetic reference models (*S*. *sp*. PCC 6803, *i*JN678 and *S*. *elongatus* PCC 7942, *i*JB785). Gram-negative properties and metabolic pathways were obtained from the template *E*. *coli* K-12 substr. MG1655 (*i*ML1515). *i*DT1294 shares 1,100 reactions with *i*ML1515 (Fig 2), which are related to transport reactions, lipid metabolism, and the production of BOF constituents. Table 1 compares the properties of the different metabolic models reconstructed for *R*. *palustris*. To our knowledge, *i*DT1294 represents the most comprehensive M-model of *R*. *palustris* available to date.

**Table 1:** Comparison of the principal model properties (reactions, metabolites, and genes) available for *R*. *palustris*.

Model	Reactions	Metabolites	Genes	Reference
*i*DT1294	2721	2123	1294	this study
Model SEED	1312	1451	980	[70]
CarveMe	1717	1260	898	[68]
Navid (*i*AN1128)	1173	1028	1128	[51]
Alsiyabi (*i*Rpa940)	1449	1541	563	[52]

#### 2.1.4. Biomass objective function

The BOF contains the principal constituents and the stoichiometry values of each metabolite involved in biomass production. The fraction of each metabolite participating in the BOF composition is defined per gram of biomass. *i*DT1294 BOF was designed employing three different sources: (1) constituents of *i*ML1515 and *i*JN678 based on their physiological similarity (Gram-negative bacteria or photosynthetic organisms), (2) estimations based on the proteomic and genomic data of *R*. *palustris* Bis A53, and (3) literature evidence of the BOF composition obtained under different experimental conditions [21]. The stoichiometric coefficients of amino acids, DNA, and RNA precursors present in the BOF were calculated based on the theoretical amino acid abundance obtained from proteomic and genomic references. The mineral fractions were determined based on mineral requirements from the literature (see Methods). Additionally, the carotenoid composition and their stoichiometric representations in the BOF were estimated assuming the presence of these metabolites in dark and light conditions [71]. The remaining constituents were estimated based on requirements of the Gram-negative model *i*ML1515. The metabolites involved in biomass production were grouped into nine principal clusters (i.e. amino acids, cell wall, pigments, cofactors, RNA, DNA, carbohydrates, minerals, and miscellaneous). *i*DT1294’s final BOF composition contains 98 constituents (excluding the theoretical metabolites representing the BOF clusters). The BOF was employed as the primary optimization objective to perform metabolic predicitions of growth and internal fluxes for the four different modes of metabolism in *R*. *palustris*.

### 2.2. Accurate growth and metabolic fluxes predictions under different experimental conditions

*i*DT1294 was validated extensively against experimental data using various carbon and nitrogen sources under different growth conditions representing the four metabolic modes (chemoheterotrophic, chemoautotrophic, photoautotrophic, and photoheterotrophic environments).

#### 2.2.1. Growth validation

*R*. *palustris* Bis A53 was grown under aerobic chemoheterotroph conditions in the dark using Biolog plates with 190 carbon (PM1 and PM2) and 95 nitrogen (PM3) sources. Growth was evaluated for 96 hours to determine which substrates were suitable nutrients for this PSNB. The growth values were normalized according to the average growth over triplicates per Biolog plate and reduced to qualitative data (growth or non-growth values). From this high-throughput phenotypic data, carbon and nitrogen compounds were mapped to *i*DT1294. Out of 190 carbon sources, 143 metabolites were identified in the M-model; carbon substrates without defined pathways for assimilation or catabolism were not mapped. Model simulations of these conditions were performed under heterotrophic aerobic conditions with ammonium and N_2_ as the preferred nitrogen sources. Mineral and O_2_ constraints were adjusted to -1000 and the final fluxes were calculated according to the carbon source limitations and BOF requirements. The carbon and nitrogen substrates employed during the phenotypic experiments are displayed in S2 Material. The Biolog plate validation results were classified and analyzed in two specific groups: carbon (PM1 and PM2) and nitrogen (PM3) compounds. For the group of carbon sources, a total of 143 compounds were mapped and analyzed using the *R*. *palustris* Bis A53 metabolic model. Carbon sources with little metabolic evidence (e.g., laminarin, 3-0-b-D-galactopyranosyl-D-arabinose, b-methyl-D-xyloside, etc.) were not included in the validation of the model. Before the gap-filling stage, the manually curated model version had 51% accuracy and Mathews Correlation Coefficient (MCC) of = 0.34 predicting carbon compound utilization. Most of the erroneous growth predictions were related to false negatives (70) since the model could not consume or metabolize different amino acids, carbohydrates, and organic acids. After the gap-filling stage, *i*DT1294 increased the accuracy to 85% and MCC to 0.68 (close to 35% more for both statistical parameters) regarding the carbon compounds. However, the growth estimations of the model were still affected by false negative predictions (19), mainly distributed in amino acids (8) and amino-containing compounds (8). The same procedure employed for the carbon set was followed in PM3 experiments to estimate the growth with 89 different nitrogen compounds. In this case, simulations were performed using sucrose as the sole carbon source. Statistical parameters (accuracy, sensitivity, specificity, positive predicted, negative predicted, and MCC) were calculated for chemoheterotrophic non-diazotrophic conditions with O_2_ uptake adjusted to nitrogen requirements. Based on the PM3 simulation results, *i*DT1294 showed the highest accuracy and modeling precision across all Biolog conditions (Table 2). Predicting the qualitative growth of nitrogen compounds, *i*DT1294 achieves an accuracy of 94%, with 100% positive predictions and MCC close to 90%. The number of false negative predictions decreased to 6 compounds distributed in amino acids (3) and amino compounds (3).

To accurately determine how *i*DT1294 modeling capabilities compare to previously published models, the same procedure was applied to the four existing GEMs (Table 1) calculating the model simulation statistical parameters. Models distinct from BiGG naming nomenclatures (ModelSEED automatic reconstruction, *i*Rpa940, and *i*AN1128) were translated to BiGG IDs to map carbon and nitrogen sources previously tested in *i*DT1294. However, from the four presented M-models, only two (*i*Rpa940 and *i*AN1128) were suitable to perform Biolog simulations under the experimental conditions employed (aerobic heterotroph dark environments). The ModelSEED and CarveMe versions did not contain the required pathways and metabolic features to grow using a sole carbon or nitrogen source under oxic, dark conditions (chemoheterotrophic). Table 2 summarizes the results for the three metabolic models.

**Table 2:** Comparative table with the statistical parameters of the three principal *R*. *palustris* metabolic models for carbon and nitrogen sources from the Biolog plates experiments (PM1, PM2, and PM3).

	*i*DT1294 PM1	*i*Rpa940 PM1	*i*AN1128 PM1	*i*DT1294 PM2	*i*Rpa940 PM2	*i*AN1128 PM2	*i*DT1294 PM3	*i*Rpa940 PM3	*i*AN1128 PM3
**True positive**	55	4	22	35	0	5	27	4	14
**True negative**	12	13	13	20	20	17	56	51	44
**False positive**	1	0	0	1	2	3	0	1	8
**False negative**	11	62	44	8	42	39	6	33	23
**Accuracy**	0.85	0.22	0.45	0.86	0.32	0.35	0.94	0.62	0.65
**Sensitivity**	0.84	0.06	0.34	0.82	0	0.12	0.82	0.11	0.38
**Specificity**	0.93	1	1	0.96	0.91	0.85	1	0.98	0.85
**Positive predicted**	0.85	1	1	0.98	0	0.63	1	0.8	0.64
**Negative predicted**	0.52	0.17	0.23	0.72	0.32	0.31	0.91	0.61	0.66
**MCC**	0.62	0.1	0.28	0.73	-0.25	-0.05	0.86	0.19	0.26

Based on Biolog validation for carbon and nitrogen sources, *i*DT1294 demonstrated better prediction capabilities for growth and substrates assimilation: a global accuracy close to 90% (50% more than *i*Rpa940, and 40% above *i*AN1128), sensitivity above 82% (almost 60% more) and MCC achieving 0.75. Most of the incorrect predictions of the previously published GEMs are based on the lack of transport reactions (S7 Material). For instance, at least 50% of the substrates from the Biolog sets were present only in the cytosolic compartment of *i*Rpa940. Regarding *i*AN1128, the M-model contains significantly more transport reactions than *i*Rpa940, although it lacks the metabolic pathways needed to employ these carbon or nitrogen sources as nutrients. Both previously published M-models contain specific information about the metabolites employed in the carbon and nitrogen validation but in the cytosol compartment. Further steps of manual refinement of the reactions transporting metabolites across the compartments and connecting the substrates to core metabolism can significantly improve the accuracy of the two metabolic models. Fig 3 displays the complete comparison of the performance of each model and the experimental results obtained from phenotyping.

#### 2.2.2. *i*DT1294 metabolic trade-offs in steady state and dynamic scenarios

Next, we tested *i*DT1294’s performance using steady state and dynamic data from the literature for diverse carbon and nitrogen sources. For steady-state data, two different sets of conditions were employed to determine the accuracy of the simulations: (1) aromatic compound degradation under anaerobic conditions in light (photoheterotroph conditions) with soluble nitrogen sources in the medium and N_2_ in the environment [7,72], and (2) consumption of organic acids and glycerol under anoxygenic photosynthesis [17].

*R*. *palustris* Bis A53 contains high-specialized enzymes capable of degrading complex aromatic compounds under anaerobic growth [2]. To exploit the growth precision of *i*DT1294 we evaluated 14 aromatic compounds (with two lignin precursors) commonly found in wastewater from chemical and food industries [7,72]. Based on the experimental growth conditions of the reference studies [7,72], the model was constraint using a single organic carbon source (i.e., aromatic substrate) for each experiment, ammonium and N_2_, and all minerals required for BOF optimization under anaerobic conditions in the light. The growth condition details employed for all experiments and simulations from the literature are summarized in S3 Material. *i*DT1294 accurately predicted the growth for 14 aromatic compounds under anoxygenic photosynthesis with 12 true positives (benzoate, benzoylformate, caffeate, cinnamate, ferulate, 3-hydroxybenzoate, 4-hydroxybenzoate, 4-hydroxybenzoylformate, DL-Mandelate, p-coumaric acid, p-coumaroyl-CoA, and vanillin) and two true negatives (protocatechuate and vanillate). For most of the carbon sources analyzed, the mechanism employed by the M-model for anaerobic catabolism of the aromatic substrates uses oxidoreductase reactions with different electron acceptors (NADH, NADPH, and FMN). The resulting metabolites from the oxidoreductases’ activity are transformed into common intermediates and integrated into core metabolic pathways (e.g., benzoate, pyruvate, and fumarate). Once *i*DT1294 was tested with the aromatic compounds using qualitative growth results, the quantitative growth rates were calculated for 1 mM substrate concentration. The predicted growth rates were compared with experimental evidence obtained from Harwood [1988] [7], shown in Table 3. The M-model predicted with an average accuracy of 84% the growth rates for all the carbon sources using ammonium or N_2_ as nitrogen sources, achieving a minimum accuracy of 74% and a maximum value above 98%.

**Table 3:** Predicted and experimental growth rates reported by Harwood [1988] [7] for *R*. *palustris* using aromatic carbon sources under anaerobic light conditions.

Compound	Experimental growth (h-1)	predicted growth (h-1)	Accuracy (%)
benzoylformate	0.0075	0.0088	83
cinnamate	0.0251	0.0310	76
4-hydroxybenzoate	0.0188	0.0140	75
4-hydoxybenzoylformate	0.0050	0.0058	84
DL-mandelate	0.0055	0.0054	99

*i*DT1294 was further validated using growth with organic acids and glycerol under anaerobic conditions in the light, employing experimental data from Govindaraju (2019) [17] to evaluate the model’s accuracy for predicting steady state and dynamic growth. Initially, the model was tested using nine different conditions with one or two carbon sources since the study was performed to establish the effect of co-substrate utilization in lactate assimilation (S3 Material). After the metabolic model was proven to successfully consume these metabolites, growth and concentration timepoint datasets from the nine experimental conditions were normalized to growth rates and consumption percentages, respectively. Subsequently, dFBA [56] was applied constraining the model with the initial biomass and substrate concentrations established by Govindaraju (2019) [17]. To calibrate the dFBA algorithm, we used the final growth rate and time point as reference data for each experiment. For the experiments with co-substrate utilization, the uptake rates for the two carbon compounds were estimated based on the exponential rate consumption of the dFBA. Regulation and inhibition features were not included in the dynamic simulation constraints. Ultimately, the correlation coefficient and exponential logarithmic parameters (R2 and log_10_P) were calculated comparing the experimental growth and consumption rates against the model estimations across the time.

*i*DT1294 demonstrates a strong correlation R2 coefficient (98.6%) for the growth rate samples. For most of the conditions analyzed, the model accurately predicted lag and exponential phase trends with clear mismatches of the starting point of the stationary phase. Additionally, substrate consumption across the conditions was predicted correctly according to the dynamic simulation time points generated through dFBA model analyses (R2>97%). In some specific cases (acetate-glycerol, butyrate and butyrate-lactate), the consumption of carbon sources was incorrectly estimated since the COBRA Toolbox algorithms assume constant uptake fluxes, ignoring the biological regulation and uptake restrictions of the organism. Surprisingly, *i*DT1294 was capable of accurately predicting growth rates after depletion of the available carbon sources. When the carbon source is depleted, *R*. *palustris* utilizes CO_2_ as the carbon source (carbon fixation). For instance, under malate and malate-lactate conditions nutrients were depleted after 100 hours and CO_2_ became the sole carbon source available; *i*DT1294 accurately predicted the change in growth rates for these scenarios. Based on the statistical correlation parameters for growth rate and substrate consumption values, our comprehensive model is established as a suitable tool to estimate and analyze the dynamic metabolic behaviors of *R*. *palustris* Bis A53 under photoheterotrophic conditions.

## 3. Discussion

### 3.1. Model reconstruction and BLASTp parameters optimization

We have reconstructed a comprehensive genome-scale metabolic model for a well-studied PNSB using semiautomatic strategies. This work represents a systems biology modeling approach to elucidate the metabolic capabilities of *R*. *palustris* under four distinct metabolic modes. We generated a high-quality, manually curated, meticulously validated metabolic model of *R*. *palustris* Bis A53. Initially, the *R*. *palustris* model was reconstructed based on three principal model references from BiGG [67] selected according to their metabolic and physiological similarities, in particular two photosynthetic organisms (*Synechocystis sp*. PCC 6803 and *Synechococcus elongatus* PCC 7942) and a well-known Gram-negative bacterium (*Escherichia coli* K-12 substr. MG1655). BLASTp parameter values were optimized by reconstructing multiple draft models employing the three template models. While there is no clear consensus on which BLASTp criteria optimize the metabolic reconstruction process, previous studies have reported similar BLASTp cut-offs to reduce the amount of false positive (wrong gene assignations) and false negative (missing genes) calls in the GPR associations [26,54,55]. However, false positive calls are negatively correlated to the number of reactions included in the draft model, leading to the dilemma of increasing the number of reactions and true positive calls along with false positive calls or reducing the number of false positive genes in the GPR associations sacrificing the total of genes correctly designated. We found the number of reactions correctly predicted as a key variable in the BLASTp parameters optimization. For selecting reactions from the template models using the RAVEN toolbox [61], the correct addition of a reaction in the model provides a higher value than a correct gene assignation in the GPR associations. This can be explained by the fact that GPR manual curation takes considerably less time than adding new reactions and properly applying quality control for each metabolic reaction. Based on the high quality of the draft model obtained using the BLASTp optimized parameters, we suggest these variables must be determined and analyzed for every draft model reconstruction due to the wide variability of the template models quality employed and the phylogenetic distance of the reference organisms. Even though there are different automatic tools capable for reconstructing draft models for bacteria, such as ModelSEED [70], KBase [73] and CarveMe [68], the resulting draft models usually contain several deficiencies and, in some cases, more than 50% of the growth metabolic predictions are inaccurate, as we highlighted in the present study.

### 3.2. Model details

Our *R*. *palustris* Bis A53 M-model contains 2,721 reactions and 2,123 metabolites associated with 1,294 genes. The resulting model was highly curated using semiautomatic and manual strategies, including GPR-specific compartment associations. *i*DT1294 was successfully validated using over 350 different carbon and nitrogen sources under several growth conditions varying O_2_ content (aerobic and anaerobic) and presence of light (light and dark), achieving the highest growth phenotype accuracy (Table 2) compared with previously published *R*. *palustris* GEMs. The validation process was performed using steady state and dynamic experimental data (Fig 4), demonstrating *i*DT1294’s capability to predict growth, consumption, and production rates under stationary conditions over time. The final M-model contains detailed metabolic pathways and features to study the most relevant subsystems of *R*. *palustris*: nitrogen fixation, denitrification, anoxygenic photosynthesis, PHB biosynthesis, H_2_ production, and aromatic compound degradation. To our knowledge, the metabolic model reconstructed, curated, and validated in the present study (*i*DT1294), is the first M-model capable to predict steady state and dynamic phenotypic data utilizing a wide range of carbon and nitrogen sources under the four *R*. *palustris* metabolic modes in three cellular compartments.

### 3.3. Model validation: Scope of the model and future aspects

*i*DT1294 accurately predicts the growth rate values of *R*. *palustris* Bis A53 using 190 carbon and 95 nitrogen sources under aerobic chemoheterotrophic dark conditions (almost 300 experimental conditions). The model contains all required reactions and constraints to simulate the BOF representing the growth of the organisms successfully. We have designed an *R*. *palustris-*specific BOF, which includes the principal metabolic constituents of a PNSB, employing experimental and theoretical calculations based on genomic and proteomic annotations. BOF optimization was tested under the four metabolic modes of this organism, demonstrating the model elasticity to precisely predict the metabolic mechanisms using the available nutrients in the environment. The resulting predictions were confirmed by experimental validation using Biolog plates (PM1, PM2, and PM3) and aromatic compounds assimilation for steady-state conditions and organic acid substrate consumption to validate dynamic growth and metabolites assimilation datasets. We estimated statistical parameters from the high-throughput phenotypic data validation, achieving values higher than 90% regarding phenotype growth accuracy. Compared to other *R*. *palustris* metabolic models, *i*DT1294 showed higher precision in true positive and negative predictions for both carbon and nitrogen sources. With this information, we identified metabolic pathways employed by this bacterium to consume different types of carbon and nitrogen compounds. Despite the "Swiss army knife metabolism", *R*. *palustris* could not metabolize several amino acids as carbon and nitrogen sources; almost 50% of the true negative growth estimations were amino acids. While the organism contains general ABC branch-amino acid transporters to transfer these amino acids inside the cell, it lacks the required enzymes to transform these amino acids into core metabolic intermediates and thereby utilize them as an energy source. In some cases (e.g., serine), the necessary enzymes and transporters can be produced by the model. The discrepancy can be explained by non-metabolic mechanisms (regulation, expression, and signaling), which are out of the scope of the metabolic model capabilities. Compared to other prokaryotic metabolic models’ accuracy, *i*DT1294 displays similar statistical parameter values for both carbon and nitrogen analyses with a global accuracy of 90% and MCC maximum value of 0.86 [26,54,59]. Furthermore, *R*. *palustris* Bis A53 M-model was curated to consume aromatic compounds under anoxygenic photosynthesis. Most of the resulting simulations achieved a quantitative accuracy close to 80%, demonstrating quantitative and qualitative precision using uncommon carbon sources. These estimations are of particular interest since *R*. *palustris* can be found in many wastewater and bioremediation consortia which contain such varied metabolites. While the M-model accurately predicted utilization of several carbon and nitrogen sources under changing O_2_ concentrations and light conditions, it still possesses some inherent limitations due to the composition behind the metabolic model. For instance, metabolic models cannot predict the partial expression of the nitrogenase complex and changes in enzyme activity due to O_2_ concentrations. These tightly regulated processes, e.g., transcriptionally, translationally, or allosterically regulated, are out of the scope of GEMs but could be partially recapitulated by including all the expression and allocation features in a subsequent model of metabolism and gene expression (ME-model) of the organism [74,75]. Finally, kinetic datasets for growth and substrate consumption are suitable for metabolic model prediction under anaerobic photoheterotrophic conditions. We determined highly correlated values for all the experimental datasets, suggesting that *i*DT1294 can be employed to understand how *R*. *palustris* Bis A53 metabolism varies over time under several different conditions.

*i*DT1294 successfully predicts qualitative and quantitative growth behavior of *R*. *palustris* under a wide range of conditions, such as with or without oxygen present, in the presence or absence of light, under molecular nitrogen-fixing conditions, in the presence of aromatic compounds, and metabolizing a variety of different carbon and nitrogen sources. Moreover, the model predicts combinations of these conditions to determine possible metabolic fluxes and pathways employed by the bacterium. Thus, *i*DT1294 represents a valuable computational tool to elucidate how *R*. *palustris* can successfully establish itself in so many different environments, such as wastewater or plant roots, and manages to metabolize varies carbon and nitrogen sources, as well as aromatic compounds, in the presence or absence of light.

## 4. Methods

### 4.1. Draft model generation

The genomic sequence of *R*. *palustris* Bis A53 was obtained from The NCBI Reference Sequence database (Refseq code: GCF_000014825.1, total proteins: 4889). Protein sequences were aligned to build the initial draft model using the BLAST algorithm for protein homology. This first draft was reconstructed using The COBRA [56] and The RAVEN [60] Toolboxes. Due to the high complexity and versatility of *R*. *palustris* Bis A53 metabolism, four reference models were selected according to the proteome comparison and metabolic capabilities from the BiGG Database [67]. Template models were divided into two groups. The first included the photosynthetic template microorganisms *Synechocystis* sp. PCC 6803, model *i*JN678 [57] and *Synechococcus elongatus* PCC 7942, model *i*JB785 [58]. The second group was integrated by Gram-negative bacteria, including template models *Escherichia coli* K-12 substr. MG1655, model *i*ML1515 [59], and *Azotobacter vinelandii* DJ, model *i*DT1278 [26]. *i*DT1278 was mainly employed as a template model to obtain the nitrogen metabolism. The initial draft model from the photosynthetic organisms and *i*ML1515 was built using optimized BLAST parameters (e-value, query length, and percentage of identity). A sensitivity analysis was performed to determine which specific BLAST parameters’ values maximize the number of true positive homology calls and minimize the number of false negative and positive calls in the Gene-Protein-Reaction (GPR) associations. The determination of the optimal values involved the assessment of each BLAST parameter and the effect of the three parameters interactions. The resulting optimal BLAST parameter values were employed to determine the GPR associations of the Gram-negative template models. Template models contained reactions associated with carbon and nitrogen fixation, photosynthesis, amino acid catabolism, lipid metabolism, nucleotide pathways, and sugar degradation. The generated draft model also contained genes from the template models, which were later removed during the model refinement stages.

### 4.2. Model refinement

Model refinement included manual curation of the GPR associations using bioinformatics databases and experimental information followed by gap-filling of new metabolic reactions not found in the BIGG Database based on genomic associations and experimental evidence.

#### 4.2.1. Manual curation

In the first step of the manual curation process, reactions in the initial draft model with exogenous proteins in the GPR associations were identified and verified in *R*. *palustris* metabolism using data from bioinformatics databases (KEGG, BioCyc, BRENDA, UniProt, and MetaNetX). Exogenous proteins assigned during the reconstruction stage were replaced with homologous sequences of *R*. *palustris* Bis A53 using the Enzyme Commission (EC) number as a reference to identify the metabolic function of the protein. For every subsystem of the model with exogenous proteins, the GPR associations were manually reviewed using KEGG, BioCyc, NCBI and PATRIC. When the sources disagreed about which protein should be associated to a reaction, a unique GPR was chosen by following the consensus among the databases and annotations. Additionally, reactions lacking any *R*. *palustris* Bis A53 proteins were checked through BLASTp. Candidate proteins from the *R*. *palustris* proteome were identified via BLAST against the proteins assigned to the GPR associations for the reaction in other microorganisms in the BiGG database. The BLASTp criteria were an e-value ≤ 1e-10, query coverage ≥ 80% and identity percentage ≥ 40%. A second step of manual curation was executed to confirm the correct assignation of the GPR associations [26,54,55]. The proteins for each reaction of the semi-curated draft model were manually reviewed based on the type of metabolic reaction, protein function, and cell compartment. Protein complexes were adjusted correctly from the GPR associations of the template organisms to the specific protein complex conformations in *R*. *palustris*. All validated reactions, metabolites, and GPR associations were distributed in three different compartments (periplasm, cytoplasm, and extracellular compartment). Metabolites were labeled according to the corresponding position in the cell. Metabolites obtained from the photosynthetic model references were carefully renamed and properly allocated to the corresponding compartment of *R*. *palustris* Bis A53. Duplicated metabolites generated during the renaming and reallocation of photosynthetic metabolites were merged and integrated into a unique metabolite identifier. Transport reactions were added using the TransportDB database [71]. Metabolite transport between compartments was curated using BLASTp. Hypothetical and putative proteins were not included in the GPR associations of the curated model to avoid false positive calls. The remaining reactions with exogenous genes in the GPR associations of the model were identified and analyzed through Flux Balance Analysis (FBA) using the COBRA Toolbox [56]. Reactions with no flux and exogenous GPR associations were removed from the M-model. Reactions with exogenous proteins carrying fluxes were maintained in the model as orphan reactions.

#### 4.2.2. Gap filling

Gap filling was performed in two steps, (1) gap filling of metabolic pathways already present in the manually curated draft model, and (2) gap filling by adding new metabolic pathways into the model obtained from different bioinformatic databases or experimental evidence from the literature.

Gap analysis was performed to recognize which compounds were disconnected in the M-model and which reactions were lacking in the analyzed pathways. Initially, disconnected metabolites were determined through dead-end analysis algorithms. The dead-end metabolites were classified according to their disconnection motive (present in one reaction, present only as a substrate or as a product). Subsequently, the involved reactions were identified to establish suitable connections of dead-end metabolites. Disconnected reactions were manually added using different bioinformatic databases (e.g., KEGG, Biocyc). After these analyses, gap filling was employed to connect pathways (lipid metabolism, gluconeogenesis, TCA cycle, etc.) through the data retrieved. Finally, dead-ends were identified through KEGG and BioCyc pathway modules and synthesized using COBRA gap filling algorithms [56]. A final test was performed to verify the correct production of each dead-end metabolite using sink reaction algorithms from the COBRA Toolbox. Subsequently, the second round of gap filling was performed to connect metabolites from the medium retrieved using literature information [17,76–79] and experimental data generated in the present study. Gap filling algorithms identified the reactions involved in the assimilation of carbon and nitrogen sources under photoautotrophic, photoheterotrophic, diazotrophic, non-diazotrophic, and heterotrophic conditions. Both aerobic and anaerobic conditions were considered to perform these simulations and the second round of gap filling. The reactions added in the gap filling process with no GPR associations were annotated as orphan reactions. Ultimately, reaction fluxes were validated using FBA to verify the predicted internal fluxes to identify the production of each metabolite present in the model.

In the second step of gap filling, new pathways were added to the refined model using semiautomatic algorithms. Specifically, carbon (aerobic and anaerobic degradation of aromatic compounds and PHB production), nitrogen (denitrification and partial denitrification), and photosynthetic (carotenoids, bacteriochlorophylls, and bacterial pigments) metabolisms and their annotation. The names of new reactions and metabolites were assigned according to the BIGG database; reactions and metabolites with no information in BiGG were added to the model according to the EC Number information (through BRENDA [64]) and bioinformatic databases (KEGG, Biocyc, etc.). Detailed information for reactions and metabolites (charge, formula, reversibility, direction, etc.) were extracted from well-reviewed biochemical databases (PubChem, UniProt, ModelSEED, KBase [73], and MetaCyc) or from the metabolic models of *R*. *palustris* Bis A53 from Machado et al 2018 and Navid et al., 2019 [51,68]. For *R*. *palustris* Bis A53-specific features, like bacteriochlorophyll biosynthesis and pathways for aromatic compound degradation, literature information using experimental evidence was employed to reconstruct these specific metabolic subsystems. Pseudoreactions with unspecific intermediates or used to represent transformations with no known pathway were not included in the M-model to avoid inaccurate predictions. All pathways of aromatic compound utilization were curated based on experimental evidence from Harwood and Ma [7,72]. A detailed list of reactions, metabolites, and genes added to the model for these pathways is presented in S5 Material. The functionalities of all reactions were validated using FBA through the model to predict biomass production. Reactions added in the second step of gap filling were tested by adding specific constraints in the model and performing simulations to measure the reaction flux distributions. Sink reaction algorithms were employed to assess the production of the new metabolites in the manually curated model.

#### 4.2.3. Final quality control and quality analysis

The final quality check was accomplished to guarantee correct GPR associations and suitably balanced reactions and metabolites. We performed *in-silico* single gene-deletion simulations (*in-silico* gene knockouts) to verify if the GPR associations are properly assigned using the COBRA and RAVEN Toolboxes. Next, we performed Mass and Charge Balance simulations of the COBRA Toolbox on the model to check for unbalanced reactions added during the model refinement. Unbalanced reactions were fixed by adding the correct formula and charge of each metabolite. Stoichiometric values assigned in every reaction were carefully reviewed and corrected for unbalanced reactions. Ultimately, the final model was analyzed by looking for ATP, NADH, and NADPH free energy production, by removing media nutrients (exchange reactions) and checking that they had zero flux.

### 4.3. Growth conditions and experimental validation

#### 4.3.1. *R*. *palustris* Bis A53 culturing and growth conditions

*R*. *palustris* Bis A53 was purchased from the American Type Culture Collection (ATCC BAA-1125). *R*. *palustris* Bis A53 was grown in mineral medium [80] in light conditions for 14 days at 26°C. Purple cultures were transferred to an aerobic medium with the following composition: yeast extract, 0.1 g; NH_4_Cl, 2.7 g; KH_2_PO_4_, 0.5 g; MgCl_2_ · 6H_2_O, 0.33 g; CaCl_2_ · 2H_2_O, 0.05 g; NaCl, 0.4 g; 9.5 ml Pfennig and Lippert’s mineral solution; and 8.5 ml vitamin solution [81]. *R*. *palustris* was cultured in aerobic medium for 3 days at 30°C under dark conditions. Optical density (OD) measurements were taken at 600 nm (OD600). A solution based on OD600 readings was prepared for the Biolog plates experiments. After the inoculation, Biolog plates were incubated aerobically at 30°C in the dark (chemoheterotrophic condition).

#### 4.3.2. Carbon source utilization assays

Bacterial suspensions of *R*. *palustris* were tested for growth on 190 different carbon sources using Phenotypic Microarray (PM) plates 1 and 2A from Biolog, Inc. (Biolog, Inc., Hayward, California) following the company’s instructions. Briefly, cell suspensions from a 10-day old culture (see above) were washed and resuspended in Biolog’s IF-0a GN/GP Base (1.2X) inoculating fluid (#72268) up to an optical density of 0.025 at 600 nm (OD600, Molecular Devices SpectraMax M3 Multi-Mode Microplate Reader (VWR, cat # 89429–536)). The suspentions were further supplemented with ATCC trace minerals and vitamin supplements (ATCC-MDTMS and ATCC-MDVS). 100 uL of these washed cell suspensions were inoculated into each well of the Biolog plates PM1 and 2A (a full list of compounds including a blank well with no carbon source (negative control) can be found here). Plates were incubated at 30°C without shaking with lids coated with an aqueous solution of 20% ethanol and 0.01% Triton X-100 (Sigma) to prevent condensation [82]. We defined substrate utilization by *R*. *palustris* as an OD600 increase > 0.02 (readout of total cell biomass from substrate use) over a 96 hour incubation period.

#### 4.3.3. Nitrogen source utilization assays

The bacterial suspensions for the nitrogen use assays were prepared in the same way as for the carbon source but were washed in IF-0a GN/GP Base (1.2X) inoculating fluid (#72268) containing 5 mM potassium phosphate monohydrate, pH 6 (Millipore-Sigma), 2 mM sodium sulfate (Millipore-Sigma) and 20 mM sucrose as the carbon source. The rest of the steps and determination of substrate utilization as a nitrogen source were similar to the ones described above for the carbon source assay.

### 4.4. Constraints and growth simulations

Experimental conditions from the literature were employed to determine specific medium constraints of the M-model. For all heterotrophic growth conditions, carbon and nitrogen uptake rates were calculated depending on the values obtained from results in the literature. For phototrophic (photoautotrophic and photoheterotrophic) conditions, photon fluxes were set to a maximum uptake rate value of 1000 and limited based on the CO_2_ exchange flux. The constraints related to mineral requirements were set according to the BOF estimations. Growth and internal fluxes simulations were performed in the COBRA Toolbox for MATLAB [56,83] using the flux balance analysis procedure [84]. The stoichiometric coefficients of the amino acids, RNA, and DNA requirements in the BOFs were set according to the genome and proteome sequences of *R*. *palustris* Bis A53 [85]. Mineral concentrations and coefficients in the BOF of the model were set according to literature information [4,80,81]. Ammonium was used as the main nitrogen source for initial heterotrophic model estimations. Acetate was set as the principal carbon source based on the experimental reports [11,17,30]. Subsequently, 24 experimental growth conditions from the literature were evaluated using FBA to identify the model accuracy and active pathways in all the conditions. Besides ammonium, other nitrogen sources were tested in the previous conditions, and N_2_ was tested as the nitrogen source (diazotrophic capabilities) in combination with other soluble nitrogen compounds for 14 anaerobic conditions [86]. The reactions involved in carbon assimilation for autotrophic and heterotrophic conditions were identified and mapped in the model. Fluxes of the reactions involved were used to build possible pathways employed by *R*. *palustris* Bis A53 under autotrophy and heterotrophy. Reaction flux distributions were analyzed to identify how pathways participate according to the medium conditions. Specifically, pathways involved in energy production, amino acid, nucleotide and lipid metabolism, pollutants degradation (aromatic compounds), photosynthesis, polymer production (PHB), carbon fixation, and cofactor and vitamin synthesis were analyzed according to the participation of each subsystem in all the different conditions. For experimental conditions with no specific uptake rates or experiments with only qualitative results (growth or non-growth), the validation process was reduced to true positive, true negative, false positive, and false negative calls. Additionally, the model was validated through experimental data generated in this study, with a total of 190 carbon sources (Biolog plates PM1 and PM2) and 95 nitrogen sources (Biolog plates PM3). For carbon source assessment, ammonium assimilation was not fixed to a specific value (non-diazotroph conditions). The experimental results from Biolog plates were matched with data retrieved from the literature to determine and evaluate model precision during the simulations. During nitrogen condition simulations, sucrose was used as the main carbon source. Statistical parameters were calculated according to the comparison between the M-model predictions and the experimental values. All Biolog plates conditions were tested and simulated in the model under chemoheterotrophic conditions (photons and CO_2_ assimilation constrain set to 0). Carbon and nitrogen sources without available or well-defined metabolic pathways for assimilation and metabolic integration were not mapped into the metabolic model. Additionally, nitrogen fixation metabolism and N_2_ assimilation constraints were fixed to 0 since the experiments were performed under aerobic conditions. The model accuracy from the Biolog plates results was compared with the *in-silico* predictions of other *R*. *palustris’* models to determine the quality of model simulations. Statistical parameters were calculated for each model to determine model precision, accuracy, sensitivity, positive and negative predictions, and MCC. These statistical parameters were calculated using the formulas employed in the confusion matrices to evaluate model performance previously described [87].

### 4.5. M-Model validation using physiological data

For in-depth validation of the *R*. *palustris* M-model, kinetic studies were employed to determine the prediction capabilities of the metabolic model. Carbon source consumption and growth rate values were obtained from the literature [17]. A total of nine growth conditions using organic carbon sources under anaerobic conditions in the light. The experimental conditions included three conditions with a single carbon source and six with two organic carbon sources. The M-model built and curated in the present work was constrained according to the initial conditions of each experiment, and growth rate values were calculated using specific step times to achieve well-defined exponential plots based on the exponential growth equation. Initial biomass concentrations were used as starting point for growth rate estimations. The dFBA algorithm was used to calculate new growth values and substrate consumptions over time. Furthermore, the metabolic model by Alsiyabi et al. (*i*Rpa940) [52] was modified to assimilate carbon sources employed in these experiments. dFBA was performed in *i*Rpa940 for all nine experimental conditions and the results were compared to our M-model predictions. Statistical parameters and correlation values were calculated for growth rates and substrate consumption data using experimental time points as a reference. Correlation values were estimated employing the Pearson correlation algorithm of Python based on the experimental observations and predicted values.

## Supporting information

S1 Material Summary table of the reconstruction and statistical parameters using different BLAST criteria for *Rhodopseudomonas palustris*. (XLSX)

S2 Material Carbon and nitrogen substrates employed for phenotyping using Biolog plates. (XLSX)

S3 Material Growth values of experimental and simulation data for multiple time points. (XLSX)

S4 Material Summary table of the reactions and metabolites added into *i*DT1294 for bacteriochlorophyll biosynthesis. (XLSX)

S5 Material Detailed list of reactions, metabolites, and genes addded to *i*DT1294 for aromatic compound degradation. (XLSX)

S6 Material Table containing the reactions with exogenous genes associations and new GPR associations based on manual curation. (XLSX)

S7 Material List of Biolog plates simulation results using *i*Rpa940 and *i*AN1128. (XLSX)
